# Contraceptive prevalence and types used among female secondary students in public schools in Obio Akpor, Rivers State Nigeria

**DOI:** 10.4314/ahs.v25i1.42

**Published:** 2025-03

**Authors:** Kelechi Favour Andrew, Anthony Okpani, Foluke Adeniji

**Affiliations:** 1 Population and Reproductive Health Division, School of Public Health, University of Port Harcourt, Nigeria; 2 Department of Obstetrics and Gynecology, University of Port Harcourt Teaching Hospital, Port Harcourt, Nigeria; 3 Department of Preventive and Social Medicine, University of Port Harcourt Teaching Hospital, Port Harcourt, Nigeria

**Keywords:** Contraceptive use, prevalence, female students

## Abstract

**Background:**

Correct and consistent contraceptive use among adolescents reduces the incidence of sexually transmitted infections and unplanned pregnancies.

**Objective:**

The purpose of this study was to determine the prevalence of contraceptive use and ascertain the types of contraceptives used by female secondary school students in Obio Akpor Local Government Area.

**Methods:**

The study used a cross-sectional design and a multistage random sampling method to recruit students who were sampled using a pre-tested semi-structured interviewer-assisted questionnaire, and the data collected was analyzed using Statistical Package for Social Science (SPSS) Version 20.0.

**Results:**

Three hundred and twelve participants were recruited for this study. The rate of contraceptives ever used by respondents was 35%; 34% had used them at their last sexual activity, while only 20% were consistently using contraceptives. Older students between the ages of 16 and 18 years used more contraceptives than the younger students (13 and 15 years), with a probability (p-value) of 0.100. Contraceptives used by respondents include condoms, post-coital pills, and withdrawal methods (88%), 24%, 8.82%, and 2.94%, respectively.

**Conclusion:**

This study revealed that the contraceptive prevalence among students was poor, and there is a serious need to increase adolescents' access to reproductive health services and information.

## Introduction

Correct and consistent contraceptive use among adolescents can help reduce the incidence of sexually transmitted infections and unplanned pregnancies.

The exuberant and explorative behavior of adolescents makes their sexual activity unprotected, with a high possibility of having multiple sexual partners, which predisposes them to contract sexually transmitted infections, HIV/AIDS, and a high incidence of pregnancy.^1^-^5^

“Unplanned pregnancy poses a major challenge to the sexual and reproductive health of young adults in the world, especially in developing countries, and thus the need to improve on the knowledge and utilization of contraceptives.”^6^ The public health importance of female adolescent sexual and reproductive health cannot be ignored, as health risks faced by the adolescent girl during pregnancy account for 15% of the global burden of disease (GBD) for maternal conditions and 13% of all maternal deaths^7^,^8^.

These unplanned pregnancies among adolescents also account for 23% of the overall burden of disease or disability-adjusted life years due to pregnancy and childbirth^9^.

According to the National Bureau of Statistics 2016 data, over 14,743 female students attended secondary school in public secondary schools in Rivers States^10^.

In addition, most of these unplanned pregnancies are among adolescents and usually end in abortion. Most of these abortion procedures are carried out in secret and in unsafe circumstances, and they lead to serious life-threatening complications^11^-^13^.

The contraceptive prevalence rate in some African countries in the northern part varies; countries like Mauritius, Morocco, Algeria, and Egypt have prevalence rates of 75%, 63%, 61.4%, and 60.3%, respectively^14^. Countries in the southern part of Africa, like South Africa, Zimbabwe, Namibia, and Swaziland, have prevalence rates of 60.3%, 60.2%, 55.1%, and 50.6%, respectively. In the West African region, they have lower prevalence rates; Nigeria has a low prevalence rate of 14%. The sexual and reproductive health of female adolescents is a very important concern in any society, and as such, there should be a conscious effort to support it.

Adolescents should be able to access comprehensive sexuality education and counseling on family planning at all times^15^.

The purpose of this study was to determine the prevalence of contraceptive use and ascertain the types of contraceptives used by female secondary school students in Obio Akpor Local Government Area.

## Methods

### Study setting

There were 16 public secondary schools with a total enrollment of 14,784 students and 9,898 female students in Obio Akpor local government area (10) While, the study was carried out in four different secondary schools in the Obio Akpor Local Government Area. Obio Akpor Local Government Area is an urban local government area in Rivers State, southern Nigeria.

### Study Design

The study used a cross-sectional descriptive study design.

### Sample Population

The sample population is comprised of female senior secondary school students in the Obio Akpor Local Government Area of Rivers State.

### Eligibility Criteria

#### Inclusion Criteria

The Inclusion criteria of the study consisted of senior secondary female students in public secondary schools in the Obio Akpor Local Government Area of Rivers State.

#### Exclusion Criteria

The Exclusion criteria excluded the female students who were not willing to participate in the study because of personal reasons.

### Sample Size Determination

The sample size was determined by Fisher's formula for calculating the sample size.

Sample size (n) = Z^2^ pq e^2^

Sample Size (n) = Z^2^ p (1-p) e^2^

Where: q = 1-p

Z = Standard normal deviate set at 1.96.

e = error margin was at 5% (0.05)

p= prevalence rate of sexual activity among female secondary students in a study 15 is 24.7%.

### Sampling Method

The sampling method that was used was multi-stage random sampling, which first involved the random selection of four schools from the different wards in the Obio Akpor local government. A total of three hundred and twelve participants were recruited. There was a random selection of three upper secondary classes from each of the schools, among the selected; one class of SS1, SS2, and SS3 was selected. The third stage was a simple random selection of the students from each of the classes that were selected in stage two. In each school visited, seven-eight (78) students were randomly selected from the senior secondary classes of SS1, SS2, and SS3. In each class that was selected, twenty-six (26) students were randomly selected from the different classes of SS1, SS2, and SS3.

A pre-tested, semi-structured interviewer-assisted questionnaire was used for data collection, which consisted of three sections on socio-demographic characteristics, sexual behavior, and use of contraceptives.

### Data Analysis

Data was collected and inputted into the Microsoft Excel spreadsheet, from where it was transferred and analyzed using Statistical Package for Social Science (SPSS) Version 20.0.

Descriptive statistics like the mean, proportion, frequency, percentages, and standard deviation were used to describe participants' socio-demographic variables, sexual activity variables, and contraceptive use, and these were made into tables for easy understanding. A p-value of less than 0.05 was statistically significant.

## Results

The general objective of the study was to determine the prevalence of contraceptive use and ascertain the types of contraceptives used by female secondary school students in the Obio Akpor Local Government Area.

A total of three hundred and twelve (312) questionnaires were given out, and[KA5] three hundred and five (305) were returned to the researcher. This indicated that seven (7) questionnaires received little to no response from the students. The results of this study showed a response rate of 97.75% among the respondents, of which 2.25% were lost to no response. The socio-demographic characteristics' of the participants were as follows: 51.08% of the respondents were between the ages of 13 and 15 years, while 48.2% of the respondents were between the ages of 16 and 18 years. SS1 and SS2 classes had 102 respondents each, which was 33.44%, respectively, while SS3 classes had 101 respondents.

All the respondents in the study were Christians and came from 15 different states in Nigeria.

Generally, the students in the senior secondary class ranged in age from 13 to 18 years. From the table above, the mean age of the respondents was 15.45 years, with a 95% confidence interval and a standard deviation of 1.33.

Results from Table 2 show the family characteristics of the respondents. About 77.05% of the participants lived with their parents, and 22.05% lived with their guardians. None of the respondents lived alone, with their friends, or with a male partner. About 84.59% of the parents of the respondents lived together, and 15.41% do not[KA6] live together. Among those respondents whose parents do not live together, 19.15% were never married, 2.13% were divorced, 55.32% were widowed, 17.02% were separated, and 6.38% were single.

**Table 2:** Family characteristics of the respondents

Characteristics	Frequency n=305	Percentage (%)
**Whom do you live with**		
Parents	235	77.05
Guardian	70	22.95
Male Partner	0	0.00
Peers/Friends	0	0.00
Alone	0	0.0
**Do your parents live together**		
Yes	258	84.59
No	47	15.41
**If no, what reason (n=47)**		
Never Married	9	19.15
Divorced	1	2.13
Widowed	26	55.32
Separated	8	17.02
Single	3	6.38
**What type of family do you have**		
Monogamous family	281	92.13
Polygamous family	24	7.87
**Were you born with your Parents married**		
Yes	271	88.85
No	34	11.15
**How do you describe your family socio-economic status**		
Poor	80	26.23
Comfortable	206	67.54
Rich	19	6.23

The respondents had 92.13% of them come from monogamous families, and the remaining 7.87% were from polygamous families. Some of the respondents, 88.85%, were born with their parents married, while 11.15% were born out of marriage.

About 26.23% of the respondents considered their family poor, 67.54% considered their family comfortable, and the remaining 6.23% thought theirs was rich.

Results from Table 3 showed that the mean age of menarche of the students was 12.57 years, with a 95% confidence interval and a standard deviation of 1.08. The mean age of sexual debut of the students was 14.07% with a 95% confidence interval and standard deviation of 1.83, with 36% of the sexually active starting by age 10–13 years, 63% by age 14–17 years, and 1% at more than age 18.

**Table 3:** Sexual Behaviour of respondents

Characteristics	Frequency n=305	Percentage (%)
**Age at first menstruation**		
10-13	253	82.95
14-17	52	17.05
*Mean ± SD*		*12.57 ±1.08*
**Have you had sex before**		
Yes	100	32.79
No	205	67.21
**Age at first sexual intercourse (n=100)**		
10-13	36	36.00
14-17	63	63.00
≥18	1	1.00
*Mean ± SD*		*14.07 ±1.83*
**Do you have a boyfriend**		
Yes	93	30.49
No	212	69.51
**How many sexual partners do you have in the last 3 months**		
One	80	26.23
Two	9	2.95
None	216	70.82
**Do you have more than one sexual partner at the same time (n=77)**		
Yes	8	10.39
No	69	89.61
**Have you ever been pregnant before**		
Yes	13	4.26
No	292	95.74
**How many times have you been pregnant (n=13)**		
1	10	76.92
2	2	15.38
3	1	7.69
**Have you had an abortion before (n=13)**		
Yes	13	100
No	0	0.00
**Whom would you consider an ideal partner**		
Schoolmate of same age	100	32.79
Young man	120	39.34
Older working male	14	4.59
No response	71	23.28

Among the respondents to the study, 30.49 % accepted having a boyfriend, and 69.51% did not have a boyfriend. The sexually active respondents had a varying number of sexual partners in the last three months, and the number varied from one to two. Still, among the sexually active, 10.39% had more than one sexual partner at the same time, and 89.61% had one sexual partner at a time.

Results further showed that 4.26% of respondents had been pregnant before. The table further showed the number of times the respondents had been pregnant. Among those respondents who admitted to having been pregnant before, 13 of them admitted to having had abortions, and the respondents' preferences in their choice of an ideal partner were varied.

Results from Table 4 indicated the last date of sexual activity among the sexually active respondents. Among the sexually active adolescents, the mean age of the sexual partners of the respondents was 23.99 years, with a 95% confidence interval and a standard deviation of 5.98.

The ages of their sexual partners varied: 40.45% had partners between the ages of 15 and 24, 31.46% had partners between the ages of 25 and 34, 4.49% had partners between the ages of 35 and 44, and 23.6% did not know the age of their sexual partner.

**Table 4:** Sexual Behaviour of sexually active respondents

Characteristics	Frequency	Percentage (%)
	n=100	
**When was the last time you had sex (n=100)**		
Last 3 days	2	2.00
Last week	7	7.00
Last Month	28	28.00
In the last 3 months	44	44.00
In the last 6 months	5	5.00
In one year	14	14.00
**Age of sexual partner (n=89)**		
15-24	36	40.45
25-34	28	31.46
35-44	4	4.49
Don't know	21	23.60
*Mean ± SD*		*23.99 ±5.98*
**Do you use any form of protection against pregnancy when engaged in sexual activity? (n=100)**		
Yes	34	34.00
No	66	66.00
**If yes, specify (n=32)**		
Condom	30	88.24
Post coital pills	3	8.82
Withdrawal	1	2.94

Among the sexually active respondents, 66% do not use any form of protection, while 34% use protection such as condoms, post-coital pills, and withdrawal methods.

Table 5 showed that the older students within the ages of 16–18 years, which was 71.43%, used more contraceptives than the younger students (13–15 years).

**Table 5:** Contraceptive use according age group

Do you use contraceptive			
Q1 AGE GRP	No	Yes	Total
**13-15**	31 (47.69)	10 (28.57)	41 (41.0)
**16-18**	34 (52.31)	25 (71.43)	59 (59.0)
**TOTAL**	65	35	100

This further showed that the level of association was not statistically significant, with a probability (p-value) of 0.100.

From Table 6 below, results showed that 35% of the sexually active respondents use contraceptives. Only 34% made use of contraceptives at their last sexual intercourse; the methods used included condoms, post-coital pills, and withdrawal methods. The condom was the method most commonly used. Most of the respondent's reasons for contraceptive use were to avoid pregnancy and prevent sexually transmitted disease. Among the 35 sexually active respondents that admitted to using contraceptives, only 20 (57.14%) of them were consistent in their contraceptive use. Those sexually active respondents that don't use contraceptives had their various reasons for not using contraceptives, as seen in the Table above.

**Table 6:** Use of Contraceptives

Characteristics	Frequency n=100	Percentage (%)
**Do you use contraceptive**		
Yes	35	35.00
No	65	65.00
**Did you use at your last sexual intercourse**		
Yes	34	34.00
No	66	66.00
**Which contraceptive was used (n=34)**		
Condom	30	88.24
Post coital pills	3	8.82
Withdrawal	1	2.94
**What was your reason of using contraceptive (n=35)**		
To avoid pregnancy	34	97.14
To prevent STDs	1	2.86
**Do you always use contraceptive method when engaged in sexual activity (n=35)**		
Yes	20	57.14
No	15	42.86
**If no, what was the reason for not using contraceptive (n=65)**		
Lack of access (availability to contraceptive)	7	10.77
Objection to its use	1	1.54
Lack of awareness	51	78.46
Too young to use it	5	7.69
Religion doesn't allow it	1	1.54

## Discussion

The purpose of this study was to determine the prevalence of contraceptive use and ascertain the types of contraceptives used by female secondary school students in Obio Akpor Local Government Area.

The low levels of contraceptive use reflect the spontaneity of adolescents' sexual activity, their lack of knowledge, and the barriers they face in obtaining contraceptives. As a result, adolescents have the problem of inconsistent and incorrect use of contraceptives^17^,^18^.

The findings on the prevalence of contraceptive use among female students revealed that the proportion of sexually active senior students in this study that had ever used contraceptives was 35%, and 34% of them used them at their last sexual intercourse. In line with two studies^16^ carried out in Port Harcourt, Nigeria, which showed that 30.4% of the sexually active students had used modern methods of contraception, and^19^ another studied both junior and senior secondary school students in Port Harcourt, it revealed that among the sexually active, 45.3% had ever used any method of contraception; the junior students made more use of contraception than the senior students, with 64.5% versus 35.5%.

In contrast to these findings, another study done in Lagos, Nigeria revealed that only 5% of female secondary school students in Lagos actually used contraceptives,^20^ and another study^22^ in Darees-Salam, Tanzania, reported 12.4% of the students made use of contraceptive methods. This result could be explained by the lack of sexual health information among secondary students, the difficulty in accessing sexual health products, or the bias in accessing contraceptives.

In a study done 21 in Northern Ghana, a trend was observed that revealed that as the age and educational level of respondents increased, there was likelihood for contraceptive use to increase. This was in line with the findings of this study; where older students used more contraceptives than younger students, this could be a result of more knowledge of the benefits of contraceptives and better access to contraceptive products among the older students.

A study 24 among adolescents in public schools in Brazil revealed that 56.1% of the students had used contraceptives within the last six months, and the prevalence was higher among young women during the last time they had intercourse. While another study^23^ among adolescents from poor neighbourhoods in Managua, Nicaragua, showed that 54% reportedly used modern contraceptives, this was in contrast to the findings of this study showed that less than half (35%) of the sexually active students used contraceptives, 34% used them at the last sexual intercourse, and 20% always used contraceptives.

This showed that adolescents from more developed countries had a higher prevalence rate of contraceptive use than adolescents from less developed countries. This could be a result of better-accessible sexual and reproductive health services that are made available to adolescents.

The findings of this study showed that the types of contraceptives used by the participants were the post-coital pill, withdrawal method, and condom, which were most commonly used. In line with the results of this study, a study carried out in Port Harcourt reported that the participants in the study^16^ used various methods of contraception, such as the Rhythm method, combined oral contraceptive pills, withdrawal method, condom, post-coital pills, injectable progesterone, diaphragm, and intrauterine device (IUD). While, this was in contrast with the study carried out^23^ in Managua, Nicaragua, adolescents used hormonal injections, oral contraceptives, and implants. Another study,^19^ where the female students used both traditional and modern contraceptives. These include condoms and oral pills; others include Andrew's liver salt, pepper soup, lemon, alcohol, club soda, 7-Up soft drink, herbs, Udah seed antibiotics, etc. This means that the adolescents used different methods of contraception that they knew and were readily available to them. The strength of this study was that it captured a snapshot of the contraceptive use among the students, and that it took less time to conduct the study. However, it did not look at possible changes over time or its possible causes. Due to the delicate nature of the questions and the setting in which the information was gathered, most participants in this study were unwilling to answer if they were sexually active for fear of being judged, which was one of its weaknesses. To acquire the right answers from them, the questions had to be formulated in various ways.

Secondly, there is also the issue of religious bias among some teachers and principals in some schools visited, which affected their attitude towards the data collection process in the study area.

## Conclusion

This study revealed that the contraceptive prevalence among the students was poor, and there is a serious need to increase adolescents' access to contraception and reproductive health services and information, as this will help avoid unplanned pregnancies, promote healthy lives and well-being, improve reproductive health, and meet the third and fifth sustainable development goals. Unwanted pregnancy among female students affects not only the girl child but her economic earning power as well as that of her entire family, and it is important to educate the girl child on how to take charge of her sexual and reproductive health.

## Recommedations

The recommendations require collaboration with the family, schools, community, health care institutions, non-governmental organizations, and the government at all levels.

Firstly, parents should create a conducive atmosphere at home that will encourage good relationships and proper communication among the children, especially the female children, and try to give their girls early sex education.

Secondly, schools have an important role to play in ensuring that sexual and reproductive health is taught in the best way possible. Students should be able to ask questions about their sexuality without having to be judged. Health care institutions should also be receptive to adolescents who need answers as regards their sexual and reproductive health (SRH).

Thirdly, the government should be able to make and implement policies that will improve the sexual and reproductive health (SRH) of the female adolescents and also re-introduce school-based sexual and reproductive health (SRH) policies, as this will help teach the adolescents about their sexuality and help them acquire skills in managing responsible decisions and actions as they affect their sexual and reproductive health (SRH). This, in turn, will help her take charge of her life and work towards improving her health.

## Figures and Tables

**Table 1:** Socio-demographic characteristics of the respondents

Characteristics	Frequency n=305	Percentage (%)
**Age**		
13-15	158	51.80
16-18	147	48.20
*Mean ± SD*		*15.45 ± 1.33*
**Class**		
SS 1	102	33.44
SS 2	102	33.44
SS 3	101	33.11
**Religion**		
Christian	305	100.00
Muslim	0	0.0
Traditional	0	0.0
**Geopolitical Zones**		
South South	170	55.74
South East	131	42.95
South West	2	0.06
North Central	2	0.06
North East	0	0.00
North West	0	0.00
